# Advancement in Leishmaniasis Diagnosis and Therapeutics

**DOI:** 10.3390/tropicalmed8050270

**Published:** 2023-05-10

**Authors:** Fernanda N. Morgado, Fátima Conceição-Silva, Maria Inês F. Pimentel, Renato Porrozzi

**Affiliations:** 1 Laboratório de Imunoparasitologia, Instituto Oswaldo Cruz, Fundação Oswaldo Cruz, Rio de Janeiro 21041-250, Brazil; 2 Instituto Nacional de Infectologia Evandro Chagas, Fundação Oswaldo Cruz, Rio de Janeiro 21040-360, Brazil; 3 Laboratório de Protozoologia, Instituto Oswaldo Cruz, Fundação Oswaldo Cruz, Rio de Janeiro 21041-250, Brazil

Leishmaniasis is a complex of clinical manifestations that affects thousands of people in the world each year according to WHO [1,2,3], and is caused by several species of protozoa belonging to the genus Leishmania. Depending on the species involved and host factors, they can produce cutaneous, mucocutaneous or visceral disease [4]. Since the last century, many groups of researchers have been dedicated to studying the parasite, pathogenic aspects of parasite–host interaction, diagnosis, treatment and prevention of the disease. Despite these efforts, there is still a lack of highly sensitive, accurate and easy-to-perform diagnostic tools, especially in areas with limited resources, which is of particular importance to overcome since many human cases occur in rural regions. The treatment of leishmaniasis is another area of study that needs improvement. The few recommended drugs are quite toxic. The availability of drugs to the affected people varies greatly in different countries and they are not always part of the national lists of medicines to be freely given to affected people [3]. Moreover, pharmaceutical industries are less interested in the research of more efficient drugs for diseases that mainly affect poor people. Thus, this special topic aims to gather and share the knowledge and experience of research groups, focusing on studies on the diagnosis and treatment of leishmaniasis in its cutaneous, mucocutaneous and visceral clinical forms. It comprises 10 papers, including that by do Socorro Carvalho Miranda et al. (2022) who studied the identification and distribution of visceral leishmaniasis in the Amazon region of Brazil from 2011 to 2020 [5]. Based on geotechnology, cases were associated with socioeconomic, epidemiological and environmental characteristics, with emphasis placed on the high prevalence in preschool children (0–4 years), and also with agriculture, mining activities and deforestation. The authors discussed the association between non-sustainable development models and the emergence of cases of visceral leishmaniasis. The paper offers an interesting discussion about the contribution of these factors to the increase in the prevalence and distribution of the disease in the affected territory, as well as the measures that should be adopted to control visceral leishmaniasis.

The development of more sensitive, specific and easy-to-perform diagnostic methods is one of the necessary measures for the control of leishmaniasis. This would allow for better, easier and faster identification of affected people and, consequently, the early implementation of specific treatment. The diagnosis of cutaneous leishmaniasis is based on the identification of *Leishmania* spp. in the lesions using parasitological methods. These include the search for amastigotes in scrapings taken from the lesion and stained with Romanowsky-type dyes, and parasite isolation from clinical samples in specific media such as NNN/Schneider biphasic medium. These are highly specific tests; however, they require appropriated equipment and infrastructure and their sensitivity may vary due to many factors such as insufficient sample quantity, improper storage, errors in smear slide preparation and a lack of technical experience of the laboratory personnel. The development of molecular methodologies applied to different types of clinical samples for the diagnosis of leishmaniasis is now being optimized to overcome all of the difficulties found in parasitological tests. Thus, Reina et al. (2022) studied the molecular identification of the Leishmania species in samples of skin lesions from patients clinically diagnosed with cutaneous leishmaniasis in Panama, but with a negative parasitological diagnosis [6]. Using the PCR-HSP70 RFLP and HSP70 sequencing methods, the species Leishmania (Viannia) panamensis and Leishmania (Viannia) guyanensis were identified. In addition, an association was demonstrated between the presence of a genetic variant and lower parasite loads that led to negative parasitological diagnoses via parasite culture and the scraping of the lesion. Based on these data, the authors reinforced the need to use molecular methods for the diagnosis of leishmaniasis in samples from patients with clinical presentation and epidemiological history suggestive of leishmaniasis, but with a negative parasite isolation or visualization. In this context, and with the aim of evaluating the sensitivity of different methods for identifying leishmaniasis in skin lesions, Ferreira et al. (2022) compared colorimetric in situ hybridization (CISH) and histopathological and immunohistochemical (IHC) techniques in samples obtained from patients with American tegumentary leishmaniasis [7]. The authors showed that the IHC and CISH techniques might also be very useful for the diagnosis of lesions with a low parasitic load. Although IHC showed greater sensitivity compared with the other techniques, it showed false positive results due to the cross-recognition of fungal cells, possibly due to the polyclonal nature of the antibody used. Hence, the authors recommended the use of CISH for diagnosis based on samples of skin lesions with a low parasitic load. On the other hand, the authors pointed out that these cross-reactions obtained in IHC could be minimized using specific monoclonal antibodies. These techniques are useful for parasitological diagnosis in the tissue sections of skin lesions.

Another interesting work by Rojas-Jaimes et al. (2022) described a PCR protocol for the detection and quantification of the small subunit of the ribosomal RNA gene from the Leishmania species inside wild ticks: Amblyomma sabanerae and Rhipicephalus microplus [8]. Although they found a high frequency of positivity in the ticks analyzed in this study, the potential role played by these ectoparasites in disease transmission and/or the parasite life cycle remains to be elucidated, as discussed by the authors.

Precise and early diagnostics are important in order to establish treatment as soon as possible. Treatment may present complexity depending on the clinical form of the disease and the presence of comorbidities. The mucosal form can lead to tissue destruction in the nasal and oral mucosa, frequently generating sequelae [2,9]. It presents exacerbated inflammation, which may evolve causing greater difficulty in achieving a clinical cure [2,9]. In this special topic, two works were published focusing on the treatment of leishmaniasis. Cincura et al. (2022) compared two treatment protocols for patients with the mucosal form: one group was treated with only pentavalent antimony, while the other group received a combination of pentavalent antimony and pentoxifylline, a TNF inhibitor [10]. The authors showed that there was an association between the production of TNF and the severity of the clinical manifestations, and that the treatment with the two associated drugs had advantages, such as faster wound healing and a lower rate of therapeutic failure. The second-treatment-focused article evaluated intralesional meglumine antimoniate as a therapy for cutaneous leishmaniasis in 152 Bolivian patients [11]. Since meglumine antimoniate has considerable toxicity that may preclude its systemic use in elderly patients or patients with comorbidities, intralesional treatment may be a safer alternative in these cases. Rojas Cabrera et al. (2022) demonstrated that intralesional treatment showed grades of efficacy and therapeutic failure very similar to the conventional treatment (systemic drug), but with fewer adverse effects and a shorter treatment time [11]. In addition, they confirmed the feasibility of training the health care professionals on the front line to treat patients. As described above, pentavalent antimony may cause important adverse effects. Unfortunately, there are few other drugs available, whereby their use may vary according to the clinical form, and at least some of them may induce relevant adverse effects. In addition, there is plain evidence pointing to the emergence of resistant strains [12]. Thus, it is necessary to develop new, effective and safer treatment options. Pitasse-Santos et al. (2022) studied the active compound 1,2,4-oxadiazoles with regard to its in vitro antiproliferative effect against trypanosomatids (*L. amazonensis* and *Trypanosoma cruzi*) and cancer cell lines [13]. Interestingly, the authors discussed the similarities in pathogenesis and the common strategies that might be used to treat these two apparently different diseases, and described the entire innovative process of the synthesis and construction of these compounds. Also, Paulini et al. (2022) obtained via a gene editing technique using CRISPR-Cas9 Leishmania donovani knockout strains for the secreted acid phosphatase protein (SAcP) [14]. Among the data presented by the authors, this virulence factor proved to be very important in maintaining the parasites in the spleen of BALB/c mice. The spleen is a secondary lymphoid organ and plays an important role in the immune response against systemic pathogens. Furthermore, it is described as an organ of parasitic persistence in visceral leishmaniasis [15]. Consequently, the study of Paulini et al. pointed out that SAcP may be a promising target for the treatment of the disease [14].

Finally, the special topic presents a systematic review of aspects of leishmaniasis in kidney transplant patients [16] and a review of the diagnosis and treatment in patients with leishmaniasis from Romania [17]. Both are important since general readers do not very often access this information.

The data published in this special topic emphasize the need for more studies on the diagnosis and treatment of leishmaniasis and reinforce that we are still far from controlling the disease. To achieve this goal, it is necessary to invest in research to develop more efficient diagnostic tools and better treatments, as well as training for health and laboratory professionals in endemic regions. We must remember that environmental and demographic changes directly affect the interaction between the parasite and its host, facilitating transmission. The more people at risk of infection, the greater the pressure for diagnostic and treatment solutions, such as those discussed in this research topic, since all of these factors have a direct impact on the emergence and re-emergence of infectious and parasitic diseases, including leishmaniasis.
